# Type 1 plasminogen activator inhibitor (PAI-1) in clear cell renal cell carcinoma (CCRCC) and its impact on angiogenesis, progression and patient survival after radical nephrectomy

**DOI:** 10.1186/1471-2490-10-20

**Published:** 2010-12-03

**Authors:** Dragomir P Zubac, Tore Wentzel-Larsen, Tomas Seidal, Leif Bostad

**Affiliations:** 1 Department of Surgical Sciences, University of Bergen, Norway; 2 Centre for Clinical Research, Haukeland University Hospital, Bergen, Norway; 3 Clinic of Pathology, County Hospital Halmstad, Halmstad, Sweden; 4 Department of Pathology, Haukeland University Hospital, Bergen, Norway; 5 The Gade Institute Section for Pathology University of Bergen, Bergen, Norway

## Abstract

**Background:**

To examine the expression of type 1 plasminogen inhibitor (PAI-1) in clear cell renal cell carcinoma (CCRCC), and its possible association with microvessel density (MVD), the expression of thrombospondin-1 (TSP-1), nuclear grade, tumour stage, continuously coded tumour size (CCTS) and to assess the value of PAI as a prognostic marker in 162 patients with CCRCC treated with radical nephrectomy.

**Methods:**

A total of 172 consecutive patients with CCRCC treated with radical nephrectomy were enrolled in the study. The expression of PAI-1, TSP-1 and factor VIII were analysed on formalin-fixed, paraffin-embedded tissues without knowledge of the clinical outcome. Ten cases, where PAI-1 immunohistochemistry was not possible due to technical problems and lack of material, were excluded. Sixty-nine patients (43%) died of RCC, while 47 patients (29%) died of other diseases. Median follow-up was 13.8 years for the surviving 46 patients (28%).

**Results:**

Nine percent of the tumours showed PAI-1 positivity. High expression of PAI-1 was significantly inversely correlated with TSP-1 (p = 0.046) and directly with advanced stage (p = 0.008), high NG (3+4) (p = 0.002), tumour size (p = 0.011), microvessel density (p = 0.049) and disease progression (p = 0.002). In univariate analysis PAI-1 was a significant prognosticator of cancer-specific survival (CSS) (p < 0.001). Multivariate analysis revealed that TNM stage (p < 0.001), PAI-1 (p = 0.020), TSP-1 (p < 0.001) and MVD (p = 0.007) were independent predictors of CSS.

**Conclusions:**

PAI-1 was found to be an independently significant prognosticator of CSS and a promoter of tumour angiogenesis, aggressiveness and progression in CCRCC.

## Background

Angiogenesis is considered essential for tumour growth and metastasis. Increased angiogenesis in different tumours, as measured by microvessel density, is a predictor of adverse prognosis [1]. Its regulation is complex and the balance between pro- and antiangiogenic factors in a given tissue microenvironment, determines the angiogenic phenotype.

Type 1 plasminogen inhibitor (PAI-1) is a serine protease catalysing the conversion of plasminogen to plasmin, and is considered an important measure of tumour vascular remodelling and neovascularisation as described in breast carcinoma [2]. Despite this contention, melanoma growth has been found to be unaffected by the levels of PAI-1 expression [3]. PAI-1 has also been found to be a potent inhibitor of cell migration and angiogenesis and therefore was thought to inhibit invasion and metastasis [4]. Furthermore, it has been suggested that PAI-1 can either enhance or inhibit tumour growth and angiogenesis depending on its concentration [5]. In several carcinomas PAI-1 expression has been found to be higher than in normal cells and associated with tumour growth, invasion, and metastasis [6,7]. Divergent results have also been reported about the clinical significance of the urokinase-type plasminogen activator (u-PA) system in renal cell carcinoma (RCC) [5,6,8-10]. Thus, the biological role of PAI-1 is complex and its clinical importance remains controversial.

CCRCC is known to be a highly vascularised tumour. We therefore hypothesized that the expression of PAI-1 might have an important role for the biological behaviour of this type of tumour and thus may prove to be of future importance for treatment.

The purpose of this study was to evaluate the occurrence and expression pattern of PAI-1 in CCRCC by using immunohistochemistry; to assess a possible association between the PAI-1 expression pattern and microvessel density (MVD), the expression of TSP-1, nuclear grade (NG), tumour stage and size; and finally to examine the impact of PAI-1 on tumour progression and cancer-specific survival (CSS) in CCRCC.

## Methods

### Patients

The patient material in this series is described in detail elsewhere [11]. Briefly, a total of 172 consecutive patients with CCRCC treated with radical nephrectomy (RN) during the years 1985 - 1994 were enrolled in the study. However, due to technical problems and lack of material, 10 cases where PAI-1 immunohistochemistry could not be performed, were excluded from the study. The specimens were examined at the Department of Pathology, Central Hospital, Karlstad, and the Department of Pathology, Haukeland University Hospital, Bergen. Tumour staging was ranked according to the 2002 TNM classification system using the American Joint Committee on Cancer (AJCC) stage grouping [12]. The nuclear grading was ranked according to Fuhrman[13] and dichotomized into a two-grade system: low-grade (Fuhrman NG 1 and 2) and high-grade (Fuhrman NG 3 and 4). Continuously coded tumour size (CCTS) was given in cm by measuring the greatest diameter.

Approval to use the biological material for research purposes was granted in 2004 by the local authority at Karlstad Central Hospital in Sweden according to Swedish regulations. In Norway the appropriate Norwegian authority, Norwegian Social Science Data Services, recognized this approval. The study was carried out in accordance with the standards of World Medical Association Declaration of Helsinki as revised in 2008.

### Follow-up

Complete information on the cause of death was available in all 162 cases. Last date of follow up was April 30^th^, 2004, and median follow up for the population studied was 5.6 years; mean 7.0 years (range 0.01-19.4 years). The median follow-up for the surviving 46 patients (28%) was 13.8 years.

### Immunohistochemistry (IHC)

Immunohistochemistry was performed on 4 μm sections from formalin-fixed, paraffin-embedded tissues. The slides were deparaffinised microwaved for 15 minutes and incubated in 60 minutes with the primary antibody: the TJA6 clone, Novocastra Laboratories Ltd. The staining procedures were performed on DAKO Tech-Mate 500 slide processing equipment. Antigen localisation was revealed by the standard avidin-biotin-peroxidase method. Harris haematoxylin was used for counterstaining. Positive and negative controls were used. Necrotic areas, prominent hyalinization, and hemorrhagic areas were excluded from the analysis. The staining procedures, analyses and antibodies used concerning MVD and TSP-1 expression have been described previously[14].

**Figure 1:**
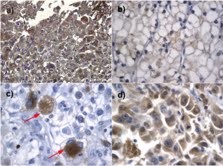
**PAI-1 antigen expression in CCRCC**. a) cytoplasmic PAI-1 staining in areas with high NG; b) weak staining in areas with low NG c) single scattered highly atypical PAI-1 positive tumour cells (arrows); d) strong expression in areas with sarcomatoid dedifferentiation

As shown in Figure 1(a, b, c, d), cytoplasmic PAI-1 reactivity was detected in RCC cells. Staining of stromal cells was not found.

### Semiquantitative analysis of PAI-1 and data processing

Screening for intratumoral hot spots under low power magnification (100× magn., Olympus BX51 microscope) revealed the areas with the highest staining intensity for PAI-1.

Ten representative images at ×400 magnification in the hot spot areas were taken by photomicroscope (Olympus U-Tvo.5×c). The expression level was quantified by computing the percentage of representatively stained surface-area in the image. The mean value for each phase fraction (intensity level) in the ten images (HPF; ×400) was calculated. The highest value determined the staining intensity level of the tumour. Tumour sections were classified as having low PAI-1 expression when they showed no or negligible/equivocal reactivity (score 0-1). Tumours with detectable PAI-1 immunoreactivity were considered to have moderate or high PAI-1 expression (score 2-3) (Figure 1a-d).

All analyses were accomplished in the AnalySIS Image Processing [Microsoft Windows NT5.0 (Build 21915) Service Pack 4]. For a better understanding of the relationship between growth and progression of CCRCC tissues that contain PAI-1 proteins, we also looked for a possible correlation to MVD (Factor VIII) and TSP-1. The Semiquantitative analysis of MVD and TSP-1 are reported elsewhere [14].

### Statistical procedures

The association between categorical baseline variables was characterized by cross tabulations and tested using exact chi-square tests for associations between dichotomized variables, and using exact Mann-Whitney tests for associations between dichotomized and ordinal or continuous variables. The associations between time to progression and baseline characteristics were investigated by univariate Cox regression. Microvessel density was dichotomized at the mean value for all 162 patients CSS, and was investigated by univariate and multivariate Cox regression. Univariate relationships were also investigated by Kaplan-Meyer analysis.

For multivariate Cox regression for CSS by PAI-1 status, adjustments were made for stage, TSP-1, nuclear grade, MVD and CCTS. Harrell's concordance index [15] was used to characterise predictive accuracy. Statistical significance was defined as p < 0.05. SPSS Statistics 17.0 (SPSS Inc, Chicago, IL, USA) and R (the R Foundation for Statistical Computing, Vienna, Austria) were used for the analyses.

## Results

According to the 2002 TNM AJCC classification, 48 tumours (30%) were pT1 stage, 24 (15%) pT2, 46 (28%) pT3, and 44 (27%) stage pT4. One hundred- and nineteen tumours (74%) were of low grade (NG1 and -2) and 43 tumours (26%) were high grade (NG3 and -4). Fourteen tumours (9%) showed cytoplasmic PAI-1 positivity. One of the PAI-1 positive tumours was stage I, two were stage II, two stage III and nine were stage IV tumours. The intensity levels and expression patterns were heterogeneous and strongest in the cells of high NG (Figure 1a). Areas of low NG were either negative or rather weak staining of PAI-1 was found (Figure 1b). In some tumours only single scattered severely atypical cells showed intense cytoplasmic positivity (Figure 1c).The strongest expression was found in areas with sarcomatoid dedifferentiation (Figure 1d). Ten (71%) of the PAI-1-positive tumours exhibited high MVD compared to 59 (42%) of the PAI-1 negative tumours (p = 0.049).

The presence of PAI-1 was inversely associated with TSP-1 expression (p = 0.046). Twelve PAI-1 positive tumours (86%) were found to have low or no TSP-1 staining, compared to 82 (57%) of the PAI-1 negative tumours. PAI-1 expression correlated with tumour stage (p = 0.008). Nine tumours with PAI-1 positivity (64%) were classified as TNM stage IV compared to 35 (24%) of the PAI-1negative tumours.

The median diameter of PAI-1-negative tumours was 7.0 cm and of PAI-1-positive tumours 9.0 cm (p = 0.014). PAI-1 expression correlated positively with NG (p = 0.002). Nine (64%) of the 14 PAI-1 positive tumours had high NG (3+4), compared to 34 (23%) of the 148 tumours with low or no PAI-1 expression.

**Figure 2:**
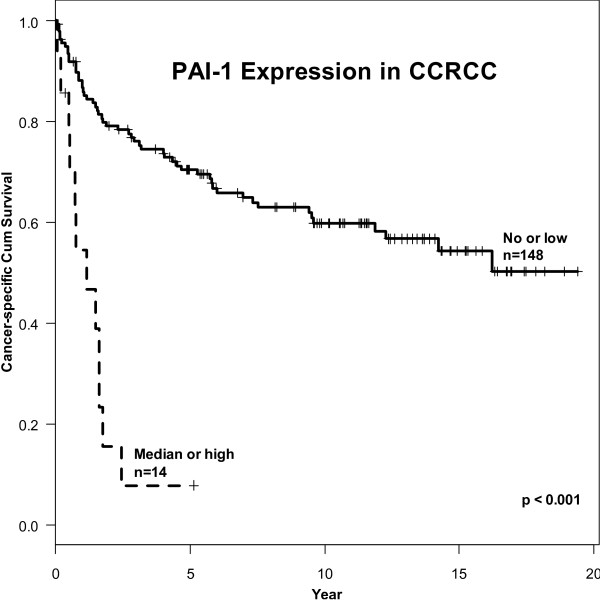
**PAI-1 expression and cancer-specific survival in 162 patients with CCRCC undergoing radical nephrectomy**.

In univariate Cox regression- and Kaplan-Meier analysis depicted in Figure 2, PAI-1 expression was a significant prognostic factor for CSS (p < 0.001) with HR of 6.49 (95% CI 3.32 - 12.68). Analyses of CSS of stage, NG, MVD and TSP-1 are reported elsewhere [14]. The multivariate analysis showed that PAI-1 expression (p = 0.02, HR = 2.32), TSP-1, stage, and MVD were independently significant predictive factors for CCS (Table 1). However, NG and CCTS were not found to be independent predictors of CSS (p = 0.341) and (p = 0.837) respectively (Table 1). Harrell's concordance index was 0.847 (0.831 after optimism correction).

**Table 1:** Cancer specific survival (CSS) and clinicopathological findings in 162 patients operated with radical nephrectomy for clear cell renal cell carcinoma; multivariate analysis

	Cancer specific survival (CSS)		
			
	HR	95% CI	p-value
PAI-1 expression			
No or low (ref.)			
Medium/High	2.92	1.15 - 4.87	0.020

TNM stage group			< 0.001
I ref.			
II	2.52	0.75 - 7.56	8.74
III	2.99	1.11 - 7.56	0.030
IV	11.60	4.27 - 31.44	< 0.001

Microvessel Density			
Low (ref.)			
High	2.19	1.24 - 3.85	0.007

TSP-1			
High (ref.)			
Low	3.69	1.74 - 7.81	0.001

Nuclear grade			
(1+2) (ref.)			
(3+4)	1.29	0.76 - 2.18	0.341

Continuously coded			
Tumour size	1.01	0.92 - 1.11	0.837

An extremely high HR for development of metachronous distant metastases in patients with PAI-1 positive tumours was found (HR = 13.71, 95% CI 2.57 - 73.14) (p = 0.002).

## Discussion

The present population-based study represents one of the largest series to date that investigates the occurrence and role of PAI-1 in CCRCC. The PAI-1 expression was found to be significantly associated with nuclear grade tumour stage and support the findings of Ohba et al. [8] but contradict the conclusions of Chautard et al.[10]. The fact that the results in different studies of RCC are somewhat diverging, may be explained by differences in the populations studied and tumour sampling. It has to be emphasized that, different methodology (cytosolic assay vs. immunohistochemical methods), has been used in these studies. Furthermore, heterogeneous distribution of PAI-1 in RCC was revealed in our study, which indicates the importance of tumour sampling. Finally, the follow-up time in our study was substantially longer.

Our findings indicate that PAI-1 positivity correlates with high MVD, and tumour growth. These findings support previous reports suggesting a possible promoting function of PAI-1 in tumour growth by its potential to modify cell adhesion [7]. The absence of host PAI-1 has been shown to prevent tumour invasion and its presence to be essential for regulation of tumour cell invasion and metastasis by promoting angiogenesis [5].

We found that the risk of progression of non-metastatic PAI-1-positive tumours was extremely high (HR = 13.71) (p = 0.002). This is in accordance with a recent study showing the overexpression of PAI-1 to be significantly associated with the presence of bone metastasis in CCRCC [16]. It has been demonstrated that PAI-1 mRNA and protein levels were dramatically increased in RCC cells expressing mutant or lacking von Hippel-Lindau (VHL) tumour-suppressor genes compared with cells expressing wild type VHL [17]. Moreover, PAI-1 has also been shown to be regulated by hypoxia inducible factor (HIF2α) [18]. In line with the findings of previous studies on various carcinomas [2] we found that high expression of PAI-1 was associated with high tumour stage and significantly lower survival rate.

PAI-1 expression occurs in CCRCC and it was found to be an independent predictor of cancer-specific survival. The expression is cytoplasmic and heterogeneously distributed and seems to be strongest in high grade tumours. PAI-1 positivity is significantly correlated with MVD, NG, tumour stage, size and progression and inversely with TSP-1. Our results show that PAI-1 independently influenced CSS of patients with CCRCC confirming the results of previous comparable studies [8,9].

The high correlation between PAI-1 expression and traditional clinical and pathological prognostic parameters like tumor stage, tumor size, nuclear grade and sarcomatoid differentiation may limit the added prognostic utility of this marker in RCC. However, our findings give strong support to the conclusion that PAI-1 may turn out to be a valuable molecular and biochemical marker for tumour aggressiveness, which can be used as a supplement or possible substitute to standard morphological methods.

Most of the tumours with PAI-1 expression were stage T3/T4. Two of the 3 patients having PAI-1 positive stage T1/T2 tumours died in RCC. Even though there were very few PAI-1 positive tumours in organ confined stages (T1/T2), this molecular marker turned out to be a highly significant predictor of developing metachronous metastasis in these patients with HR = 13,71 (p = 0.002). Further studies, however, seems necessary to fully reveal the prognostic impact of PAI-1 expression in T1-T2 tumours.

## Conclusions

We conclude that PAI-1 may have a significant role in angiogenesis, tumour growth and progression in CCRCC and may be used as a valuable molecular marker in prognostic nomograms for CCRCC. Our study indicates that illuminating the biological role of PAI-1 in CCRCC may lead to the development of new therapeutic modalities to control angiogenesis and tumour progression.

## Competing interests

The authors declare that they have no competing interests.

## Authors' contributions

DPZ was involved in conceiving the study, carried out acquisition of data and wrote the manuscript. TWL performed the statistical analysis and interpretation of data.

LB conceived the study and supervised the immunohistochemical and histopathological analysis of tumour specimens. LB and TS have been involved in drafting and revising the manuscript critically. All authors read and approved the final manuscript.

## Pre-publication history

The pre-publication history for this paper can be accessed here:

http://www.biomedcentral.com/1471-2490/10/20/prepub
